# Vorinostat unmasks MAEL to enhance DC vaccine-induced CTL killing in hepatocellular carcinoma, potentiated by TIGIT checkpoint inhibition

**DOI:** 10.1007/s00262-026-04410-2

**Published:** 2026-05-19

**Authors:** Can Liu, Qianru Zhang, Jingjing Zhang, Wenpeng Liu, Shengya Geng, Jiandong Zhang

**Affiliations:** 1 Clinical Laboratory, The Third Hospital of Hebei Medical University, Shijiazhuang, 050011 People’s Republic of China; 2 Chemical Drug Laboratory, Hebei Institute for Drug and Medical Device Control, Shijiazhuang, 050000 People’s Republic of China; 3 Hepatology department, The Third Hospital of Hebei Medical University, Shijiazhuang, 050011 People’s Republic of China

**Keywords:** MAEL antigen, Peptide, Cytotoxic T lymphocyte, Vorinostat, TIGIT blocking

## Abstract

**Background:**

To address the limited immunogenicity and immune evasion in hepatocellular carcinoma (HCC), this study developed a combinatorial immunotherapy strategy combining antigen-specific vaccination, epigenetic modulation, and TIGIT-targeted checkpoint blockade.

**Methods:**

Bioinformatic screening identified the cancer-testis antigen MAEL as a target. HLA-A*02:01-restricted MAEL peptides were used to pulse monocyte-derived dendritic cells (DCs), which primed cytotoxic T lymphocytes (CTLs). The CTL cytotoxicity against HCC cells was tested. HCC cells were treated with the HDAC inhibitor vorinostat to enhance MAEL expression, and its impact on CTL killing was evaluated. The triple combination (MAEL specific CTLs + vorinostat + anti-TIGIT) was tested in HCC xenograft mouse models, with analyses of tumor growth, survival, and immune infiltration.

**Results:**

MAEL was confirmed as an HCC-associated antigen with restricted normal tissue expression. MAEL peptide-pulsed DCs generated potent CTLs with cytotoxicity against HLA-A*02:01⁺ HCC lines. Vorinostat upregulated MAEL expression, enhancing CTL killing (*p* < 0.01). In vivo, the dual combination (MAEL specific CTLs + vorinostat) outperformed monotherapies, reducing tumor growth and prolonging survival. The triple combination achieved the strongest anti-tumor effects, with significant regression and extended survival, via increased activated MAEL specific CD8⁺ T cell infiltration and enhanced CTL effector functions (elevated IFN-*γ*, TNF-*α*).

**Conclusion:**

This triple combination strategy synergistically enhances HCC immunotherapy. Vorinostat induces MAEL expression to “unmask” tumors, while TIGIT blockade overcomes T cell exhaustion, amplifying antigen-specific CTL activity. This approach shows promise for HCC treatment.

## Introduction

Hepatocellular carcinoma (HCC) is the sixth most common cancer globally and the third leading cause of cancer-related death [1]. In China, HCC ranks fourth in the incidence of all malignant tumors but second in mortality, with approximately 64% of patients diagnosed at advanced stages, losing the opportunity for curative surgery [2]. Although surgical resection and liver transplantation remain the primary treatments for early-stage HCC, the 5-year postoperative recurrence rate is as high as 70%, and the response rate of advanced patients to traditional chemotherapy and radiotherapy is less than 20% [3]. Immune escape, tumor microenvironment suppression, and tumor heterogeneity are key factors leading to the failure of existing treatments. In recent years, the application of immune checkpoint inhibitors (such as anti-PD-1/PD-L1) and targeted drugs (such as sorafenib and lenvatinib) has significantly improved the survival of some patients, but their single-agent objective response rate (ORR) is still limited to 15%–30%, and the problem of drug resistance is prominent [4, 5]. For example, although atezolizumab combined with bevacizumab extended the median overall survival (OS) of advanced HCC patients to 19.2 months, more than 50% of patients experienced disease progression within one year of treatment [6]. Therefore, developing new tumor antigen-targeted strategies combined with immune modulation to overcome immune escape is a key direction to improve the efficacy of hepatocellular carcinoma immunotherapy.

Precision screening of tumor-associated antigens (TAAs) is the basis for developing efficient immunotherapy [7]. Traditional antigens such as alpha-fetoprotein (AFP) and Glypican-3 (GPC3) have been widely studied due to their low expression in normal tissues, but their immunogenicity is limited and their correlation with prognosis is weak [8, 9]. In recent years, through high-throughput database screening and functional verification, MAEL (Maelstrom) has gradually entered the research field as a new tumor antigen [10]. MAEL was initially identified as a germ cell-specific gene involved in the processing of piwi-interacting RNA (piRNA) and transposon silencing [11]. Recent studies have found that MAEL is abnormally activated and highly expressed in various solid tumors, and its overexpression is closely related to tumor proliferation, metastasis, and poor prognosis. For example, MAEL promotes Esophageal Squamous Cell Carcinoma Progression by activating the Akt1/RelA/IL8 signaling pathway [12]. In addition, MAEL promotes tumor metastasis through regulation of FGFR4 and epithelial-mesenchymal transition in epithelial ovarian cancer [13]. However, the immunogenicity of MAEL as a tumor antigen and its expression characteristics on the surface of hepatocellular carcinoma cells still need further verification.

Histone deacetylase inhibitors (HDACi) regulate gene transcription by inhibiting HDAC activity and increasing histone acetylation levels. In recent years, the role of HDACi in tumor treatment has attracted much attention. They can not only directly induce tumor cell apoptosis and cycle arrest but also enhance tumor antigen expression and immunogenicity through epigenetic regulation. For example, HDACi can upregulate the expression of stress ligands such as MICA/B, enhancing the recognition and killing ability of natural killer (NK) cells [14]. In addition, HDACi can also promote antigen presentation and T cell activation by regulating the maturation and function of dendritic cells (DC) [15]. Vorinostat (SAHA), the first approved HDACi, has shown anti-tumor activity in various hematological tumors and solid tumors. In hepatocellular carcinoma, vorinostat can reduce tumor cell proliferation and invasion ability by inhibiting the activity of HDAC2 and HDAC3 and enhance the killing effect of NK cells [16]. However, the regulatory mechanism of vorinostat on MAEL antigen expression is still unclear. Based on the epigenetic regulation characteristics of HDACi, it is speculated that vorinostat may upregulate MAEL expression by de-repressing histone deacetylation in the promoter region of the MAEL gene, thereby enhancing the immunogenicity of tumor cells.

T cell immunoglobulin and ITIM domain protein (TIGIT) is a newly emerging inhibitory checkpoint mainly expressed on the surface of T cells, NK cells, and regulatory T cells (Treg). TIGIT inhibits the activation of T cells and NK cells and promotes the formation of an immunosuppressive microenvironment by binding to ligands CD155 (PVR) and CD112 (NECTIN-2) [17]. In cutaneous melanoma, high expression of TIGIT is associated with functional exhaustion of tumor-infiltrating lymphocytes (TILs) and poor prognosis [18]. Preclinical studies have shown that blocking TIGIT can significantly enhance the killing activity of CTLs and NK cells and reverse the immunosuppressive microenvironment [19]. Preclinical studies have shown that anti-TIGIT monoclonal antibodies (such as tiragolumab) can significantly enhance the killing activity of CTLs and produce a synergistic effect with PD-1 inhibitors. The recent MORPHEUS-Liver clinical trial showed that the triple therapy of tiragolumab combined with the A + T regimen increased the ORR from 11 to 43%, extended the median OS to 28.9 months (vs. 15.1 months), and did not increase grade 3 or above adverse reactions [20]. This result suggests that TIGIT blockade may become a key strategy to overcome PD-1 resistance.

This study focuses on the immunotherapy of hepatocellular carcinoma. The MAEL antigen was screened through a database, and polypeptides were designed and synthesized based on its amino acid sequence to activate CTL by inducing DC cells. At the same time, HDACi Vorinostat was combined to increase the expression of MAEL antigen in hepatocellular carcinoma cells, and further combined with TIGIT blockade to enhance the killing effect of CTLs. Our research is expected to overcome multiple challenges in hepatocellular carcinoma immunotherapy and provide new ideas for clinical translation.

## Materials and methods

### Data sources and preprocessing

The RNAseq dataset containing hepatocellular carcinoma and its corresponding normal adjacent tissues were downloaded from the TCGA database. The limma package in R software was used for alignment and expression calculation of raw RNAseq data sequences. By comparing the expression profile data of tumors and paired adjacent tissues, t tests were used to screen for differentially expressed genes (DEGs). Using log2|fold-change|> 1 and p_adjust < 0.05 as thresholds to filter differential genes. The selected DEGs were subjected to functional enrichment analysis using GO (Gene Ontology) and KEGG (Kyoto Encyclopedia of Genes and Genomes) databases. Weighted Gene Co-expression Network Analysis (WGCNA) package (R package, version 1.71) was used to construct a co-expression network based on the protein-coding genes.

### Epitope peptide prediction and synthesis

Based on the HLA peptide-binding prediction algorithms provided by the BIMAS (http://www.bimas.cit.nih.gov/molbio/hla_bind/), SYFPEITHI (http://www.syfpeithi.de/bin/mhcserver.dll/epitopeprediction.htm), and IEDB (http://tools.immuneepitope.org/mhci/), HLA-A2-restricted peptides derived from the MAEL antigen were predicted. The predictive scores of these peptides met the scoring thresholds of all three prediction programs (NetCTL-1.2 score > 0.85, SYFPEITHI score > 20, and IEDB rank < 3.5). All peptides were synthesized by Fmoc solid-phase synthesis, purified by high-performance liquid chromatography (purity > 95%), and their molecular weights were confirmed by mass spectrometry.

### HLA-A2 binding affinity and stability

T2 cells were used to evaluate the binding ability and stability of MAEL-derived peptides with the HLA-A*02:01 molecule. T2 cells were stripped in PBS for 2h, washed, and resuspended in serum-free culture medium. The cells were pulsed with 80 µg/mL mutant peptides and 3 µg/mL *β*2-microglobulin. Subsequently, the cells were washed, stained with PE-conjugated HLA-A*02:01 monoclonal antibody (BioLegend), and analyzed by flow cytometry. The mean fluorescence intensity (MFI) at 0h was set as 100%, and the time required for the MFI to decrease to 50% of the 0-h value was defined as the dissociation complex 50% time (DC50). DC50 was calculated as follows: (MFI at 0h—MFI at 2, 4, or 6h)/MFI at 0 h × 100%. The binding affinity was calculated as: Fluorescence Index (FI) = (MFI of the peptide group—MFI of the peptide-free dissolution buffer group)/MFI of the peptide-free dissolution buffer group.

### Induction of peptide-specific CTLs

Peripheral blood mononuclear cells (PBMCs) were isolated from HLA-A*02:01 positive healthy donors by Ficoll density gradient centrifugation. All donors provided written informed consent prior to participation. CD14⁺ cells were then sorted from PBMCs using a CD14⁺ cell isolation kit. The CD14⁺ cells were co-incubated with GM-CSF (100 ng/ml) and IL-4 (50 ng/ml), with the medium changed every 12h and supplemented with GM-CSF and IL-4. On day 5 of culture, LPS (10 ng/ml) and TNF-*α* (20 ng/ml) were added, and the cells were further cultured until day 8 to harvest mature DCs. MAEL peptides (10 µM) were added to the mature DCs and incubated at 37 °C for 24h to obtain antigen-loaded DCs. CD8 + T cells were separated from PBMCs of HLA-A*02:01 healthy donors by Human CD8 + T Cell Isolation Kit. CD8 + T cells were co-cultured with peptide-loaded DCs (CD8 + T cells to DC ratio was 10:1). rhIL-2 (20 ng/mL) was added on the 2nd day, and the culture medium was changed half every 2–3 days. On days 7 and 12 of culture, the same proportion of peptide-loaded DCs was added for enhanced stimulation. Cultured to day 15, the cells were collected as effector T cells (CTLs). Monoclonal antibodies used for DC and CTLs analysis included CD80, CD86, CD1a, CD83, CD11c, CD69, andCD25.

### Immunopeptidomics

For the identification of MAEL-derived peptides presented by HLA-A*02:01, a recombinant antigen presentation model was established in this study. PLC/PRF/5 cells were co-transfected with pcDNA3.1 ( +) vectors encoding HLA-A*02:01 and full-length MAEL. Cells were harvested 48h post-transfection. HLA class I-peptide complexes were isolated by immunoaffinity purification using the W6/32 monoclonal antibody. Complexes were subjected to acid elution, and the bound peptides were desalted via C18 solid-phase extraction. The purified peptide mixture was separated by nanoflow liquid chromatography and directly infused into a mass spectrometer for analysis, employing a positive-ion mode nanospray ionization source. Acquired mass spectrometry data were processed using Proteome Discoverer software (version 2.4). Database searching was performed against the Swiss-Prot database via the SEQUEST HT search engine, with the false discovery rate set to < 1% and peptide length restricted to 8–12 amino acids. Finally, NetMHCpan 4.1 was utilized to predict the binding affinity of the identified peptides to HLA-A*02:01, enabling the selection of high-affinity candidate peptides.

### Induction of peptide-specific T cells from HLA-A/Kb Tg mice

Peptides (200 µg) combined with CpG-ODN 1826 (50 µg) were injected into the back of HLA-A/Kb Tg mice once a week for three weeks. The normal saline (NS) group was immunized only with CpG ODN 1826 once a week for three weeks. Five days after the last injection, spleen lymphocytes were isolated and cultured at the concentration of 5 × 10^6^ cells/mL in RPMI 1640 medium. These cells were induced by the same peptides (10 µg/mL) that were used for in vivo stimulation for a week. Intracellular cytokine staining assay and cytotoxic assay (E: T ratios = 10:1, 20:1, 40:1) were performed after a week induced in vitro.

### Cytotoxicity assay

HepG2, HCC-LM6, SK-HEP-1, PLC/PRF/5 cells were counted and plated in 96-well plates as target cells for 24h. Specific CTLs were then co-cultured with target cells at a certain ratio in normal medium for 48h. The specificity was measured by the CCK-8 assay. HepG2 and PLC/PRF/5 cells were each divided into three groups: the experimental group (shMAEL), the negative control group (shCtrl), and the blank control group. Cells in the experimental group were transduced with lentivirus expressing MAEL specific shRNA to establish stable knockdown, while the negative control group received lentivirus carrying non-targeting scrambled shRNA. Cytotoxic T lymphocyte (CTL) killing activity was subsequently assessed.

### Enzyme-linked immunosorbent assay (ELISA)

DCs and CD8 + T cells co-cultured supernatant at day 3 and day 12 was taken, and content of IFN-γ in culture supernatant was detected by human IFN-*γ* ELISA kits (Cloud-Clone Corporation, Wuhan, China). Except for blank control, each well was mixed with 50 μL supernatant or different concentrations standards and 100 μL HRP labeled antibody. After incubation at 37 °C for 60 min, the plate was washed five times. 50 μL of substrate A and substrate B were added, respectively, and it was incubated at 37 °C for 15 min. Next, 50 μL stop solution was added and the absorbance value (OD450) was measured within 15 min. Standard curve was drawn according to standard substance concentration and OD value, and IFN-γ concentration in supernatant was calculated.

### Cell culture and treatment

HepG2, HCC-LM6, SK-HEP-1 and PLC/PRF/5 cells were purchased from Cell Bank of Chinese Academy of Sciences. Cells were cultured in RPMI-1640 containing 10% fetal bovine serum. For the culture of primary liver cancer cells, hepatocellular carcinoma tissue was collected and washed with PBS after operation. The tissue was placed in P/S solution (penicillin, 5 × 10^5^ U/l; streptomycin 100 mg/l) for 45 min. Then, the tissues were cut into pieces (1 ~ 5 mm^3^) and placed into collagenase I solution (5 × 10^3^ U/l) at 37 °C for 30 min. The dissociation solution was filtered through a 150 mesh cell sieve and centrifuged for 10 min, and then the supernatant was discarded. The precipitate was resuspended in RPMI-1640 medium supplemented with 5% FBS. 2.5, 5.0 or 10.0. µM vorinostat were added to the cell culture medium, and the cells were cultured at 37˚C for 72h.

### Blocking assay

The CD8 + T cells were placed in RPMI-1640 complete medium containing 10% FBS and cultured overnight at 37 °C, 5% CO_2_. At the same time, inoculate logarithmic growth hepatocellular carcinoma cells into a 24-well plate, with 5 × 10^4^ cells per well, and culture overnight. The next day, the CD+8 T cells were havested and incubated separately with anti-TIGIT monoclonal antibodies (MG1131) and isotype controls at room temperature for 4h. After that, 5 × 10^5^ CTL cells were added to each well containing adhered cacer cells and incubated at 37 °C for 12h. Finally, flow cytometry analysis was performed. Cytokine assay and cytotoxic assay were performed.

### In vivo tumor model

BALB/c nude mice were purchased from SPF Biotechnology Co. Ltd. (Beijing). The experiments were approved by the Ethics Committee of the Third Hospital of Hebei Medical University. Nude mice were subcutaneously inoculated with 2× 10^6^ PLC/PRF/5 cells diluted in 200 µl of PBS on the right flanks. The tumors were measured every 5 days using a Vernier caliper, and the tumor volumes were calculated using the following formula: tumor volume (mm^3^) = 1/2 × length × width^2^. Once the tumor volume reached about 50 mm^3^, the mice were divided into different groups (n = 6) randomly. Meanwhile, the mice were injected with 100 mg/kg vorinostat. Anti‑TIGIT antibody(Clone 1G9) and CD8 + T cells (1 × 10^7^) generated from HLA-A/Kb Tg Mice following the above-mentioned procedure were transferred via tail vein 3 days after vorinostat administration. Similarly, 100 µl of PBS were injected into the control group. Tumor sizes were measured and calculated volumes to create growth curves. After 4 weeks of treatment, pentobarbital sodium was injected intraperitoneally to remove eyeballs for collecting blood, and then mice were euthanized.

### Statistical analyses

Data were expressed as means ± standard deviations (SD). Statistical analyses were performed using SPSS 20.0 software. For comparisons of individual data points, a two-tailed Student’s t-test (unpaired t test with Welch’s correction) was applied for parametric data, while the Mann–Whitney U test was used for nonparametric data. Additionally, effect sizes with 95% confidence intervals (CI) were reported to quantify the magnitude of observed differences, and multiple comparison control (e.g., Benjamini–Hochberg [BH]/false discovery rate [FDR] correction) was implemented to mitigate false positive errors associated with repeated testing. P value of less than 0.05 was considered statistically significant.

## Results

### Upregulation of MAEL expression in hepatocellular carcinoma

**Fig. 1:**
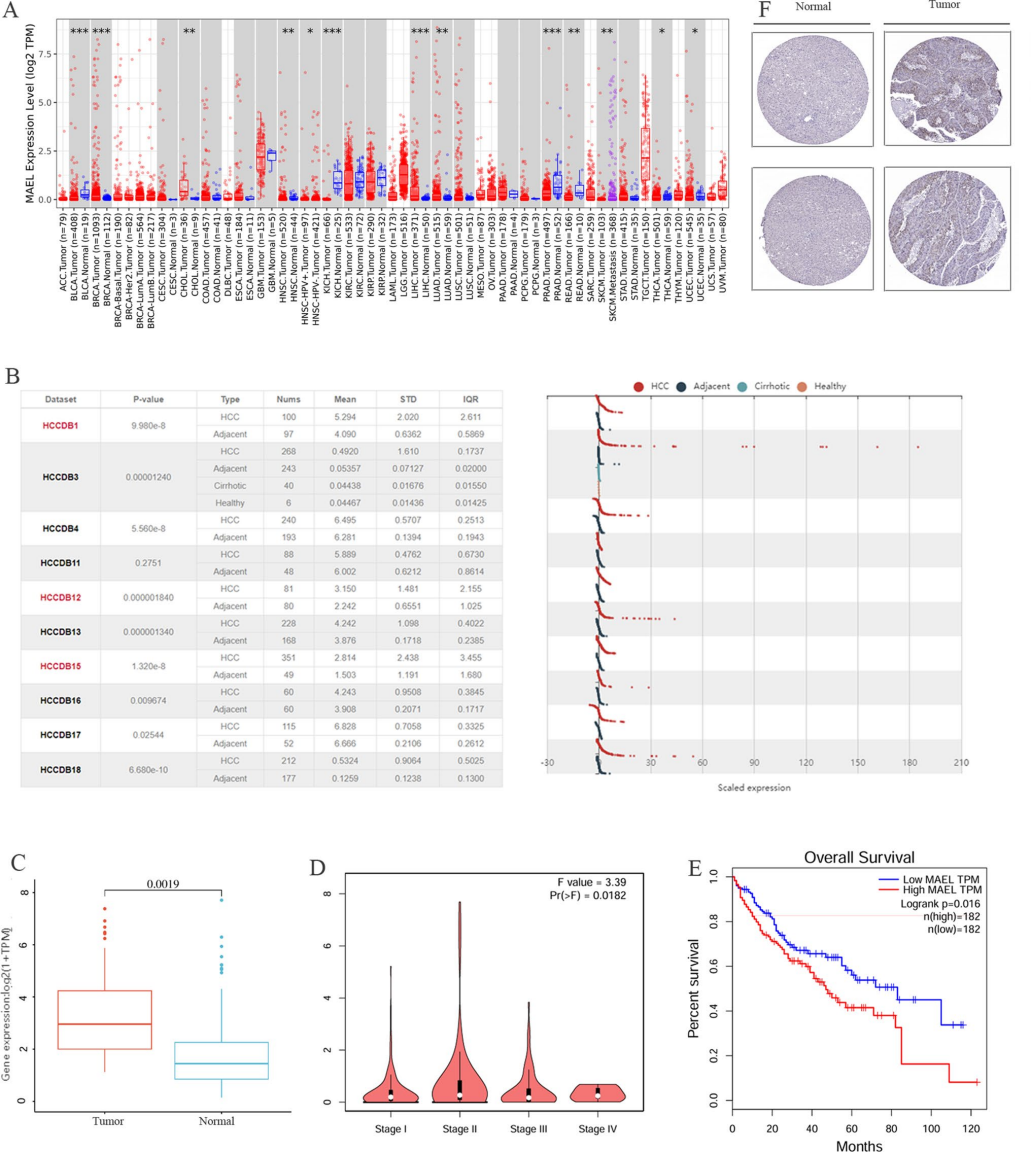
Expression level of MAEL in hepatocellular carcinoma **A** The fluctuating expression pattern of MAEL in pan-cancer. **B** Analyzing the expression level of MAEL in HCC in HCCDB database. **C** TPM data derived from the TCGA dataset. **D** The correlation between MAEL expression level and clinical stage of HCC patients. **E** The correlation between the increase of MAEL level and the survival of HCC. **F** Representative images of MAEL immunohistochemical staining in normal liver and HCC tissues. The scale is 100 μm. *P < 0.05

Using TIMER database to study the pan-cancer expression of MAEL. As shown in Fig. 1A, the expression level of MAEL is significantly increased in most cancers, including bladder urothelial carcinoma, cholangiocarcinoma, low-grade glioma, prostate adenocarcinoma and hepatocellular carcinoma. The mRNA level analysis of MAEL in various hepatocellular carcinoma cohorts from HCCDB database once again confirmed the upregulation of MAEL in hepatocellular carcinoma (Fig. 1B). In addition, the level of MAEL mRNA from TCGA data set all indicated that the level of MAEL mRNA in hepatocellular carcinoma was increased (Fig. 1C). The immunohistochemical staining results of MAEL obtained from the Human Protein Atlas (HPA) database showed that the staining intensity was higher in hepatocellular carcinoma, which further proved that the expression of MAEL was upregulated in hepatocellular carcinoma (Fig. 1F). There was no significant correlation between MAEL expression level and pathological stage of patients with hepatocellular carcinoma (Fig. 1D). Kaplan–Meier survival curve was used to explore the correlation between MAEL expression and prognosis of patients with hepatocellular carcinoma. The results showed that the high expression of MAEL was related to the shorter overall survival (OS) of patients with hepatocellular carcinoma (Fig. 1E). These data demonstrate that the expression of MAEL is significantly associated with the prognosis of hepatocellular carcinoma and may be a potential biomarker.

### Prediction, synthesis, and selection of HLA-A*0201 restricted epitopes from MAEL antigen

Given that MAEL is a potential target for tumor immunity, we then screened possible key peptide epitopes. First, we obtained the complete amino acid sequence of MAEL from NCBI, and analyzed the sequence using SYFPEITHI、BIMAS and IEDB.

To predict the potential HLA-A^*^0201 restricted epitopes. The higher scores indicate stronger binding to HLA-A^*^0201 in SYFPEITHI and BIMAS. And rank% was used to depict binding status in IEDB, in which the rank% of predicted peptide less than 0.5 was defined as strong binder. Based on these, twenty candidate peptides were selected and synthesized for further study (Table 1).

**Table 1:** Prediction of MAEL epitopes restricted to HLA-A*0201

NO	Amino acid sequences	Position in the protein	SYFPEITHI score	BIMAS score	IEDB Percentile rank
1	FVQEKIPEL	14	25	296	0.5
2	ELRRRGLPV	21	18	189	3.3
3	GMLVPKQSV	86	24	227	0.5
4	SLKSDQALL	103	23	215	0.7
5	LLGGIFYFL	110	25	336	0.4
6	EIGCIKYSL	138	18	120	2.2
7	SLQEGIMAD	145	21	174	2.0
8	IMADFHSFI	150	22	180	2.2
9	ELGHDQATV	187	21	116	0.6
10	NLYRFIHPN	198	17	271	3.1
11	CLKRMARAL	226	20	203	2.8
12	LLTVEDLVV	242	22	268	1.7
13	DILFCALAV	290	20	179	2.9
14	CISNSLATL	305	26	391	0.2
15	ATLFGIQLT	311	17	70	1.1
16	QLTGAHVPL	317	23	266	0.3
17	SVTPKMVVL	333	20	156	2.6
18	ATGDYPSGV	371	19	174	3.6
19	KISGQNSSV	380	23	302	0.3
20	GITRLLESI	392	23	225	0.8

**Fig. 2:**
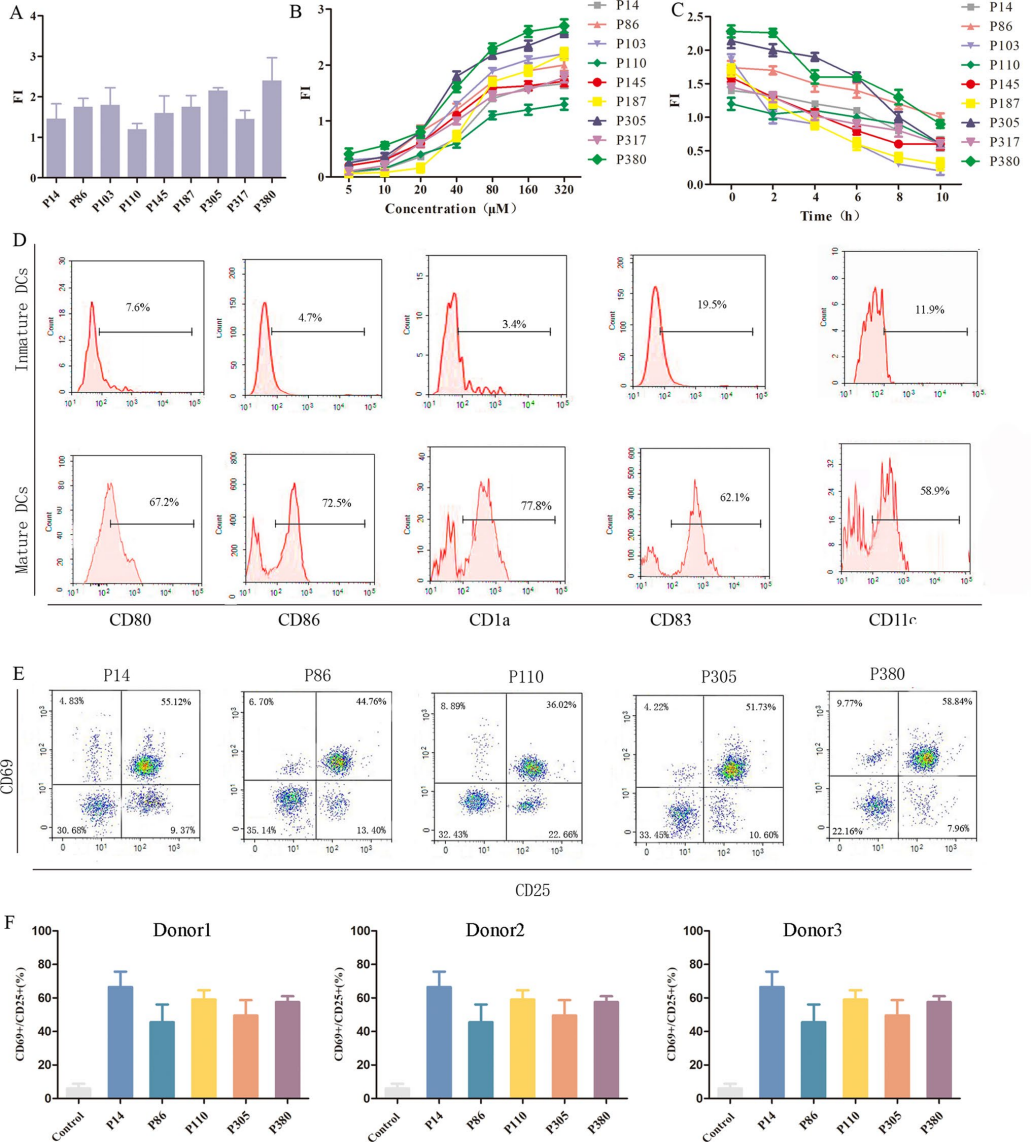
Identification and functional evaluation of HLA-A*0201-Restricted MAEL-Derived Peptides **A** Binding affinity of candidate peptides to HLA-A*0201. Fluorescence intensity (FI) values from T2 cell binding assays are shown. Peptides P14, P86, P103, P110, P145, P187, P305, P317, and P380 demonstrated high binding affinity (FI > 1), with P380 showing the highest affinity (n = 3). **B** Concentration-dependent binding of peptides to HLA-A*0201. Dose–response curves show the binding affinity of selected peptides at varying concentrations (5–320 μM), indicating a positive correlation between peptide concentration and binding affinity (n = 3). **C** Stability of peptide/ HLA-A*02:01 complexes. Decay curves show the stability of peptide/ HLA-A*02:01 complexes over time. Five peptides (P14, P86, P110, P305, and P380) formed stable complexes with a half-life (DC50) > 4h (n = 3). **D** Maturation of dendritic cells (DCs). Flow cytometry analysis of surface markers (CD80, CD86, CD1a, CD83, and CD11c) shows significantly increased expression in DCs, confirming successful DC maturation (representative of three independent experiments). **E** Activation of CD8^+^ T cells. Flow cytometry dot plots show CD69 and CD25 expression on CD8^+^ T cells co-cultured with peptide-loaded mature DCs. Peptides P14, P86, P110, P305, and P380 induced CD69^+^/CD25^+^ T cell populations ranging from 36 to 59% (representative of three donors). **F** Quantification of CD8^+^ T cell activation. Bar graphs show the percentage of CD69^+^/CD25^+^ CD8^+^ T cells induced by each peptide across three HLA-A*0201.^+^ healthy donors. All selected peptides demonstrated robust T cell activation capacity (n = 3 donors)

We then used the T2 binding test to evaluate their ability of binding affinity and stability to HLA-A*0201 molecules. Results of the binding affinity were shown in Table 2 and Fig. 2A. Peptide P14, P86, P103, P110, P145, P187, P305, P317 and P380 exhibited higher binding affinity to HLA-A*0201 (FI > 1), among which peptide P380 showed the highest binding affinity. A concentration-dependent affinity was also observed (Fig. 2B). The stability of peptides binding to HLA-A*0201 molecules was reflected by DC50. As shown in Table 2 and Fig. 2C, 5 of the 9 high-binding affinity peptides formed stable peptide/HLA-A*0201 complexes (DC50 > 4h). As an alternative strategy for identifying MAEL-derived T cell epitopes, HLA class I peptides were eluted off a MAEL- and HLA-A02:01-positive cell line to screen for the processed and presented peptides of full-length MAEL. For this experiment, five MAEL-derived peptides restricted by HLA-A*02:01 were successfully identified (Table 2). All five peptides had been validated during the preliminary selection process through in silico prediction and HLA stabilization assays, and were selected for further analysis. Additionally, according to these results, Peptides P14, P86, P110, P305, and P380 were selected.

**Table 2:** Data of ESI–MS and the HLA-A*0201 binding affinity and stability of the MAEL epitopes

Name	Amino acid sequences	Position in the protein	FI^a^	DC 50^b^	ESI–MS[M + H]^+^		Immuno- peptidomics
					Calculated (Da)	Observed (Da)	
P14	FVQEKIPEL	14	1.46	4–6h	1101.61	1102.27	_+_
P86	GMLVPKQSV	86	1.74	6–8h	957.53	958.18	_+_
P103	SLKSDQALL	103	1.86	< 2h	973.54	974.11	
P110	LLGGIFYFL	110	1.21	4–6h	1041.59	1042.27	_+_
P145	SLQEGIMAD	145	1.59	2–4h	962.44	963.06	
P187	ELGHDQATV	187	1.70	< 2h	968.46	969.01	
P305	CISNSLATL	305	2.14	4–6h	920.46	921.07	_+_
P317	QLTGAHVPL	317	1.45	2–4h	934.52	935.08	
P380	KISGQNSSV	380	2.28	6–8h	918.48	918.99	_+_

Furthermore, the ability of candidate peptides to induce CD8 + T cells in vitro was evaluated. DC-peptide co-culture experiments were carried out using healthy donor’s PBMCs. Immature DCs were induced with cytokines from adherent PBMCs of three HLA-A*02:01 + healthy donors. The results showed that the proportion of mature DC surface marker expression (CD80、 CD86、CD1a、CD83、CD11c) was significantly increased compared with the immature group (Fig. 2D), Mature DCs were successfully obtained. The mature DCs were loaded with antigen peptide, and further co-incubated with sorted CD8^+^ T cells to induce CTLs. The marker molecules of CTLs activation were detected by flow cytometry. As demonstrated in Fig. 2E, F, CD69 + /CD25 + cells accounted for 36%- 59%. These results show that peptide-specific T cells were successfully induced.

## Cytotoxic activity of peptide-specific CTLs

**Fig. 3:**
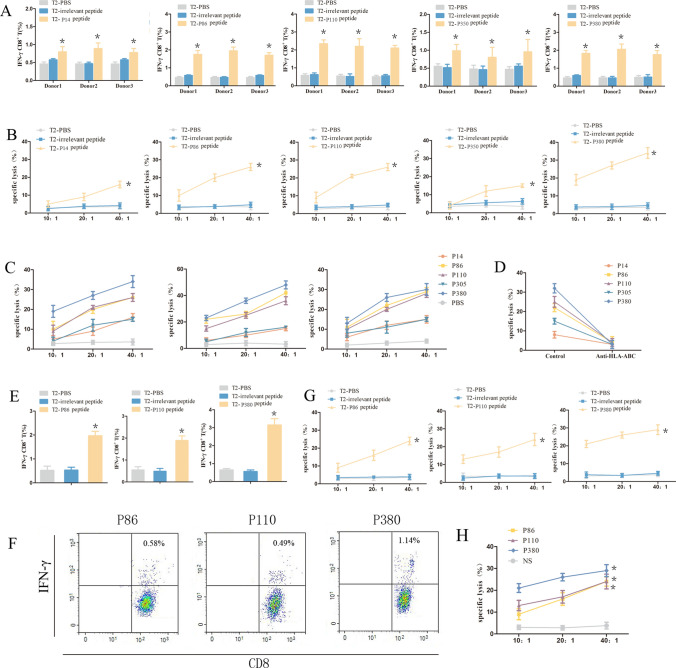
Evaluation of Anti-tumor Effects of MAEL Peptide-induced Cytotoxic T Lymphocytes (CTLs) **A** IFN-*γ* release by peptide-specific T cells co-cultured with T2A2 cells loaded with MAEL or irrelevant peptides. All five MAEL peptide-induced T cell groups showed elevated IFN-*γ* levels compared to irrelevant controls (*p < 0.05, n = 3). **B** Cytotoxicity assay showing MAEL peptide-induced CTLs lyse peptide-pulsed T2A2 cells at various effector/target ratios. Cytotoxicity was low against irrelevant peptide-loaded cells (*p < 0.05, n = 3). **C** Comparative cytotoxicity of CTLs induced by different MAEL peptide against T2A2 cells. **D** IFN-γ production in splenocytes from HLA-A2/Kb Tg mice immunized with MAEL peptides. CTLs from P86, P110, and P380 groups released IFN-γ, with P380 showing the highest response (*p < 0.05, n = 5). **E** Flow cytometry analysis of IFN-γ^+^CD8.^+^ T cells in P86, P110, and P380 groups. Representative dot plots indicate the proportion of activated CD8 + T cells. **F** Cytotoxicity assay showing CTLs from immunized mice specifically lysed MAEL peptide-loaded T2A2 cells but not irrelevant peptide-loaded cells (*p < 0.05, n = 5). **G** Comparison of lysis rates among CTLs induced by P86, P110, and P380 peptides. P380-induced CTLs exhibited higher cytotoxicity across all effector/target ratios (*p < 0.05, n = 5)

To further investigate whether CTLs induced by candidate peptides can exert anti-tumor effects, peptide-specific T cells were co-cultured with T2A2 cells loaded with MAEL peptides to detect IFN-*γ* release and lysis cytotoxicity. Results in Fig. 3A show that all five of these MAEL peptide- induced T cells could release IFN-*γ*, of which the IFN-*γ* release of each T2A2 loaded with MAEL peptide from the five MAEL peptide group was higher than that of T2A2 irrelevant group. In the cytotoxicity assay, CTLs induced by all five peptides could efficiently lyse corresponding MAEL peptide-pulsed T2A2 cells at series E/T ratio, while the cytotoxicity activity of those CTLs induced by MAEL peptide to irrelevant peptide-pulsed T2A2 was low (Fig. 3B), CTLs induced by peptide P86, P110, P380 showed a high cytotoxic activity (Fig. 3C), therefore, P86, P110, P380 were selected for vivo experiments. In addition, the cytotoxic activity of CTLs was completely abrogated by the addition of an anti-HLA-ABC antibody (Fig. 3D). These results show that the CTLs induced by MAEL peptide were MAEL peptide-specific and HLA-dependent.

To determine whether the five validated peptides can induce T cell responses in vivo, HLA-A2/Kb Tg mice were used. After immunization of the HLA- A2/Kb Tg mice with MAEL peptides at the base of the tail, the lymphocytes from the spleen were collected to detect the CTL response. T2A2 loaded with MAEL peptide were served as target cells. CTLs stimulated by the P86, P110, P380 could release IFN-γ, P380 group displayed a higher proportion of IFN-γ + CD8 + T cells (Fig. 3E, F). In cytotoxicity assay, we found that CTLs induced by MAEL peptides recognized T2A2 loaded with MAEL peptide but not T2A2 loaded with irrelevant peptide (Fig. 3G). CTLs induced by P380 peptides displayed higher lysis rates compared with the P86 and P110 peptide group (Fig. 3H).

### Vorinostat treatment promotes MAEL expression in tumor cells and enhances the killing effect of MAEL specific CTLs

**Fig. 4:**
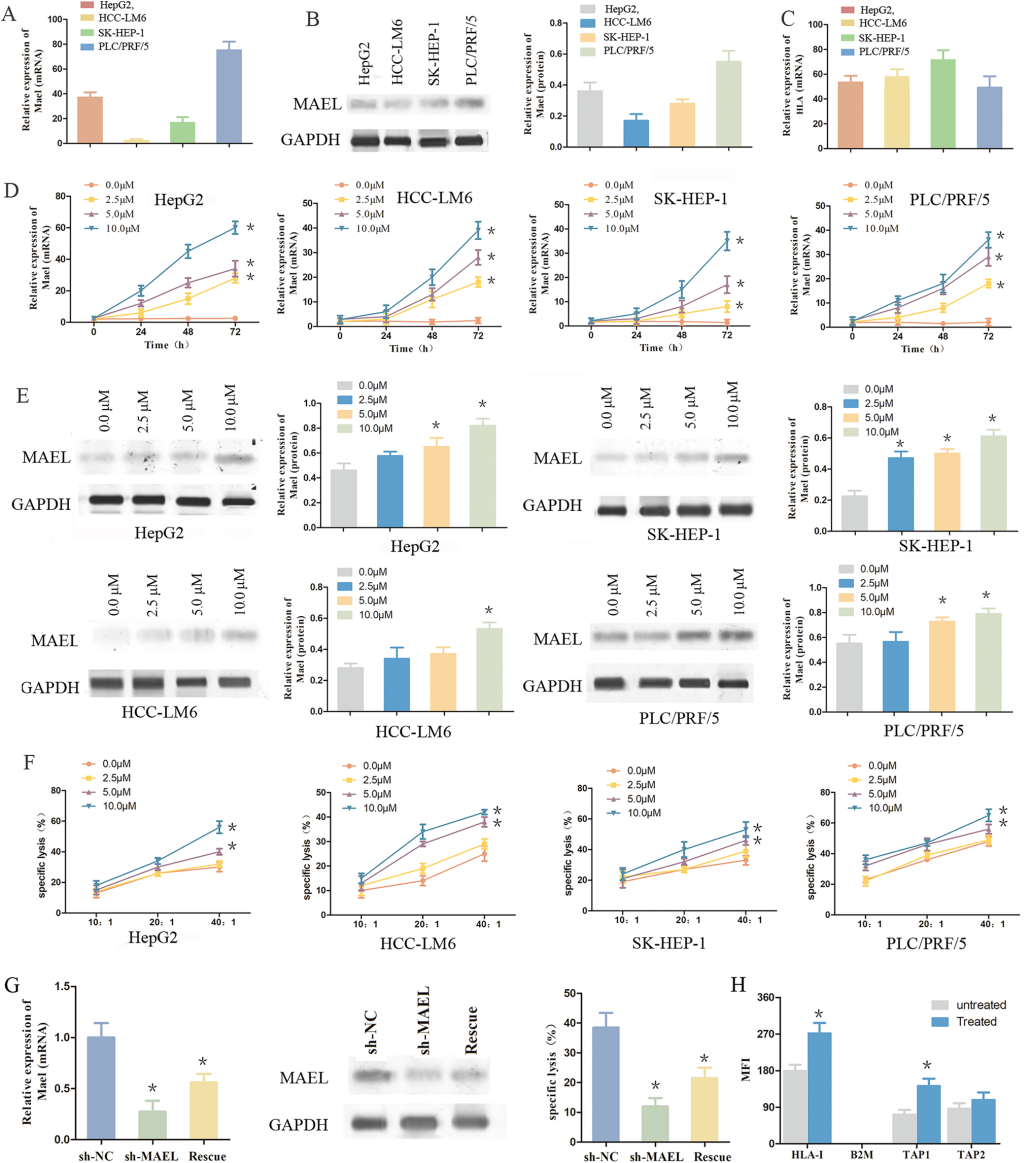
Vorinostat Upregulates MAEL Expression and Enhances CTL-Mediated Cytotoxicity **A** MAEL expression levels in liver cancer cell lines were detected by Real-time PCR. **B** MAEL expression levels in liver cancer cell lines was detected by Western blot. **C** HLA-A typing analysis of liver cancer cell lines was confirmed by flow cytometry. **D** Effects of vorinostat on MAEL gene expression (*p < 0.05, n = 3). **E** Effects of Vorinostat on MAEL protein expression (*p < 0.05, n = 3). **F** Vorinostat enhanced cytotoxicity of P380-induced CTLs. **G** The influence of MAEL gene knockdown and rescued on immune-mediated cell killing. **H** Flow cytometric analysis of the effect of Vorinostat on the expression of HLA-I, B2M, TAP1, and TAP2

The increased expression of tumor antigen in tumor cells can enhance the recognition and killing of antigen-specific T cells. Therefore, it is important to increase the expression of MAEL through some measures to combat tumors. First, we analyzed MAEL expression in hepatocellular carcinoma cell lines (HepG2, HCC-LM6, PLC/PRF/5) and a liver sinusoidal endothelial cell line (SK-HEP-1) by real-time PCR and western blotting. The results showed that the MAEL was highly expressed in HepG2 and PLC/PRF/5 cells but was expressed at extremely low levels in the HCC-LM6 and SK-HEP-1 cells (Fig. 4A, B). HLA-A2 expression was detected in all cells. As shown in Fig. 4C, the HepG2, HCC-LM6, SK-HEP-1, PLC/PRF/5 were all HLA-A2 positive. Vorinostat was chosen to induce the expression of MAEL and thus augment the cytotoxicity of CTLs. The expression level of MAEL increased with rising concentrations of vorinostat and longer treatment durations, indicating that the upregulation of MAEL by vorinostat was dose- and time-dependent (Fig. 4D). We then selected three concentrations to further induce protein expression, as shown in Fig. 4E. To further explore the role of increased expression of MAEL on immune responses of specific CTL against tumors. Hepatocellular carcinoma cells treated with vorinostat were co-cultured with P380-induced CTL, the cytotoxic activity was examined by CCK-8 assay and IFN-γ release ELISA assay. CCK-8 assays showed that P380-induced CTL showed cytotoxicity against hepatocellular carcinoma cells treated with vorinostat. With the vorinostat concentration increasing, the lysis percentages were augmented at different E/T ratios (Fig. 4F). The level of MAEL was higher in PLC/PRF/5 cells than that in other cells. Thus, PLC/PRF/5 cells was selected as the model for MAEL knockdown. After transfecting with sh-MAEL, the level of MAEL was reduced in PLC/PRF/5 cells, we found CTL-mediated specific killing was inhibited in sh-MAEL group, importantly, this suppression can be rescued upon restoration of MAEL expression (Fig. 4G). Concurrently, we assessed the expression of HLA-I, B2M, TAP1, and TAP2 in tumor cells. Vorinostat treatment led to a marked upregulation of HLA-I and TAP1. While a modest increase in TAP2, no significant alterations were observed (Fig. 4H).

**Fig. 5:**
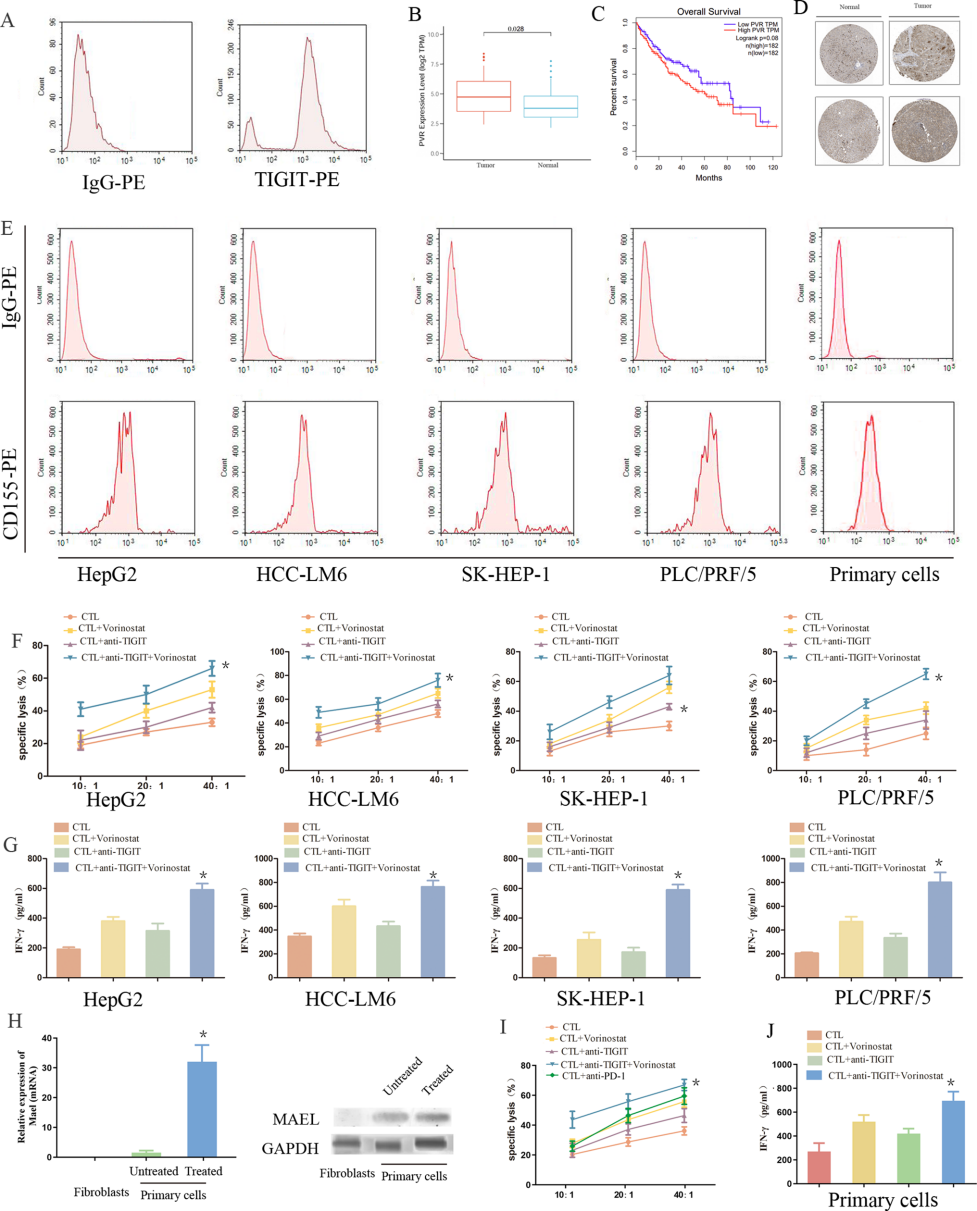
Effect of TIGIT blockade on the cytotoxicity of MAEL Specific CTL cells A. The expression of TIGIT on CTL cells was detected by flow cytometry. **B** The mRNA expression of CD155 (PVR) was analyzed from HCC tissues and non-tumor tissues in the TCGA database (n = 374 tumors vs. 50 normal; unpaired Student’s t-test; effect size: Hedges’ g = 1.2, 95% CI: 0.9 to 1.5). **C** Kaplan–Meier plot for overall survival of high and low CD155 expression groups in the TCGA database. **D** Representative images of CD155 immunohistochemical staining in normal liver and HCC tissues. The scale is 100 μm. E. The expression of TIGIT in HCC cell lines (HepG2, HCC-LM6, SK-HEP-1, and PLC/PRF/5) and primary cells was detected by flow cytometry. **F** Cytotoxic activity of CTL cells was detected by CCK-8. **G** The release of IFN-γ in HCC cell lines was detected by ELISA. **H** MAEL expression in primary cancer cells was detected by PCR and Western blots. **I** CCK-8 was used to detect the cytotoxic effect of CTL cells on primary cells. **J** The release of IFN-*γ* in primary cancer cells was detected by ELISA. Data are presented as mean ± SD from three independent experiments (n = 3)

### TIGIT/CD155 axis blockade increases the immune response of MAEL specific CTL against tumors

To assess whether immune checkpoint blockade may improve CTLs response, we firstly examined CD155 expression on hepatocellular carcinoma, and TIGIT expression on CTL by flow cytometry. The results showed that TIGIT was positively expressed on CTL cells (Fig. 5A). Compared with non-tumor tissues, the expression of CD155 in tumor tissues was significantly elevated in the HCC tissues (Fig. 5B). The Kaplan–Meier analysis indicated that there is no obvious correlation between the expression of CD155 and overall survival (Fig. 5C). The protein expression of CD155 was upregulated in hepatocellular carcinoma (Fig. 5D). The expression of CD155 was also detected in HCC cell lines and primary cells (Fig. 5E).

Then the effects of blockade of the TIGIT/CD155 regulatory axis on the cytotoxic function of CTLs were evaluated. CTLs were incubated with anti‑TIGIT antibody before co-culture with tumor cells. Cytotoxic activity was analyzed by CCK-8 assay. The result showed that TIGIT blockade enhanced the immune response of specific CTLs compared with that of the group not treated with the TIGIT blockade. The IFN-*γ* release assay results were in concert with the results. Subsequently, the effects of anti-TIGIT monoclonal antibody alone and in combination with vorinostat on the cytotoxic function of CTLs were also compared. The specific lysis rate of the CTL + anti-TIGIT + vorinostat group was significantly higher than that of the other group (Fig. 5F, G). Primary liver cancer cells were applied to further observe the killing effects of triple treatment. Human primary cells derived from human liver cancer tissue were cultured for four weeks. The expression of MAEL was positive in the primary liver cancer cells (Fig. 5H). Likewise, Vorinostat and TIGIT blockade enhanced the cytotoxicity of specific CTLs to the primary liver cancer cells, compared with that of the group not treated with the vorinostat and TIGIT blockade. Subsequently, we compared the therapeutic efficacies of PD-1/PD-L1 blockade and TIGIT blockade. Our data demonstrated that both PD-1 blockade and TIGIT blockade were capable of enhancing the tumor-killing activity of cytotoxic T lymphocytes (CTLs), with no statistically significant difference detected between the two treatment modalities (Fig. 5I). Increased levels of IFN-*γ* release were detected following culture of specific CTLs with primary liver cancer cells in vorinostat and TIGIT blockade groups (Fig. 5J). These results were consistent with the trend observed in HCC cell lines. In summary, the combination of anti‑TIGIT antibody and vorinostat enhanced the killing effect of CTLs on HCC cells.

### Vorinostat combined anti-TIGIT enhanced the immune response of MAEL specific CTL in vivo

**Fig. 6:**
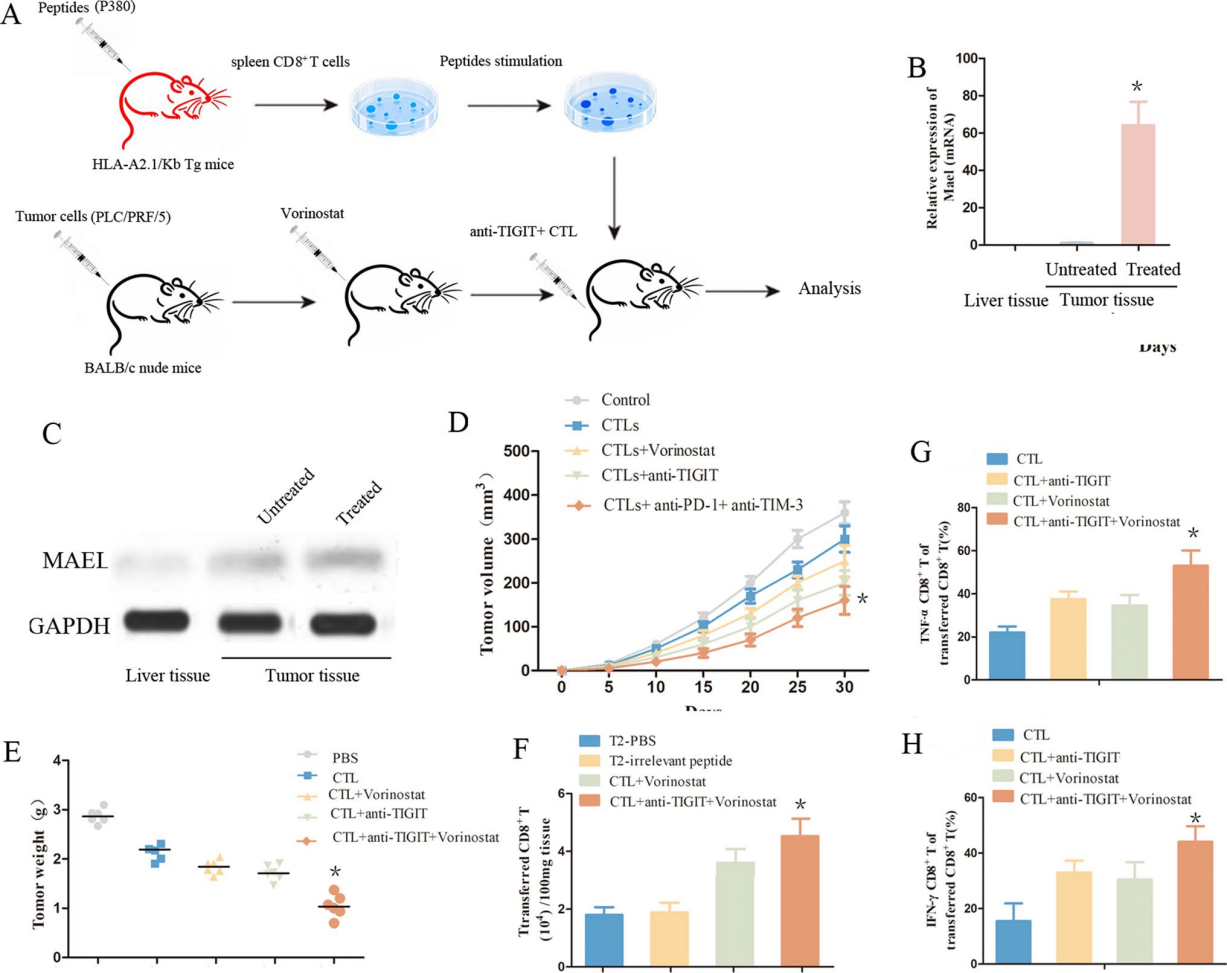
Evaluate the inhibitory effect of MAEL specific CTL cells combined with Vorinostat and anti-TIGIT antibodies on tumors in vivo **A** The BALB/c nude mice, bearing PLC/PRF/5 tumor cells, were randomly divided into five groups (n = 6/group). MAEL Specific CTL cells from the spleen of HLA-A2.1/Kb Tg Mice were re-stimulated and transferred to tumor‐bearing mice. **B** Real-time quantitative PCR detection of MAEL expression in tumor tissues and changes in MAEL expression after Vorinostat treatment. **C** Western blot was used to detect the expression of MAEL in tumor tissues and the changes in MAEL expression after treatment with Vorinostat. **D** Tumor size was measured every 5 days and shown as mean ± SEM. Student’s t-test was applied and *P* value was labeled. **E** Tumor tissues were removed from mice when mice were sacrificed. The tumors were weighed. Statistical analysis of tumor weight was determined by Student’s t-test. **F** The infiltration of specific CTL cells in tumors was analyzed by flow cytometry. **G**–**H**: The production of IFN-*γ* and TNF-*α* by MAEL specific CTL cells infiltrated in tumor tissues was analyzed by flow cytometry. *P < 0.05

Consequently, to determine the efficacy of MAEL specific CTL cells in vivo, Balb/c nude mice were inoculated with 2 × 10^6^ PLC/PRF/5 cells. After 7 days, Activated MAEL specific CTLs with or without anti-TIGIT antibodies were transferred into tumor‐bearing mice via the tail vein (Fig. 6A). MAEL specific CTLs were isolated from HLA-A2.1/Kb Tg Mice after immunization and re-stimulated with P380 for 7 days in vitro. The expression of MAEL in tumor tissues was positive, Treatment of CTL cells with vorinostat enhanced the expression of MAEL (Fig. 6B and C).

The tumor size was measured every 5 days to calculate tumor volume. In the groups transplanted with MAEL specific CTLs treated with or without vorinostat and/or anti-TIGIT antibodies, the tumor volume was significantly decreased compared with the PBS control group. In addition, the tumor volume was further reduced in mice transplanted with CTLs treated with vorinostat and anti-TIGIT antibodies compared with mice in all the other groups (Fig. 6D). Combination treatment greatly inhibited the weight of tumors (Fig. 6E). Further analysis showed that the infiltration potential of intratumor MAEL specific CTLs was significantly increased in the presence of vorinostat and anti-TIGIT antibodies (Fig. 6F). Similarly, the vorinostat and anti-TIGIT enhanced IFN‐*γ* and TNF‐*α* production in adoptively transferred MAEL specific CTLs (Fig. 6G and H). Collectively, these results indicate that vorinostat and anti-TIGIT may improve the therapeutic effect of adoptive T cell therapy and have a synergistic effect.

## Discussion

This study confirmed through database screening and functional validation that MAEL is highly expressed and immunogenic in hepatocellular carcinoma (HCC) cells, with negligible expression in normal liver tissues—characteristics that render it an ideal tumor-associated antigen (TAA). Compared with traditional antigens such as alpha-fetoprotein (AFP), MAEL exhibits significantly enhanced immunogenicity, inducing significantly enhanced cytotoxic T lymphocyte (CTL) killing activity against HCC cells without cross-toxicity to primary hepatocytes. This result aligns with previous reports on MAGE antigens, which demonstrated a 25%–30% enhancement in CTL activity via dendritic cell (DC) vaccines, underscoring the therapeutic potential of cancer-testis antigens (CTAs) in solid tumors [21]. The strong prognostic correlation of MAEL suggests its utility as a predictive biomarker for HCC immunotherapy. Future dynamic monitoring of MAEL expression via exosomal RNA sequencing may enable evaluation of vaccine efficacy. Notably, MAEL expression in HCC exhibits heterogeneity: low expression in tumor tissues of some patients may compromise CTL recognition efficiency. Therefore, immunohistochemical or genetic screening for MAEL-high patients is essential in clinical applications to improve treatment response rates. Although low expression in normal tissues theoretically poses a risk of immune toxicity, no significant off-target effects were observed in this study, indicating high safety of MAEL as a therapeutic target. Additionally, HLA restriction must be optimized for MAEL peptide design. While this study focused on HLA-A02:01-restricted epitopes, the high frequency of HLA-A24:02 alleles in Asian populations necessitates the development of multi-epitope vaccines to achieve broader population coverage [22].

As a histone deacetylase inhibitor (HDACi), vorinostat enhances histone acetylation by inhibiting HDAC activity, thereby promoting gene transcription [23]. This study revealed that vorinostat significantly upregulates MAEL mRNA and protein expression in HCC cells, potentially via deacetylation of histone H3 and H4 at the MAEL gene promoter region. Specifically, HDACi alleviates repression of the MAEL promoter by inhibiting HDAC activity, enhancing transcriptional activation. Vorinostat may also indirectly regulate MAEL expression through other epigenetic modifications (e.g., DNA methylation), though this mechanism requires further validation. Beyond upregulating MAEL, vorinostat enhances anti-tumor immunity via multiple pathways: It promotes DC maturation and migration to strengthen antigen presentation [24], while suppressing regulatory T cell (Treg) function to reduce immunosuppressive microenvironment formation [25]. These effects synergize with MAEL-targeted therapy to enhance CTL killing efficiency. However, Vorinostat use carries potential risks, such as cell cycle dysregulation and toxicity to normal tissues [26, 27]. Clinical applications therefore require precise control of dosage and treatment duration to balance efficacy and safety [28]. Developing more specific, efficient, and low-toxic HDACis or combination regimens represents a critical future research direction.

TIGIT, a key inhibitory molecule in the tumor immune microenvironment, is highly expressed in HCC and correlates with poor prognosis [29, 30]. To investigate the role of TIGIT in anti-tumor immunity, a humanized anti-TIGIT antibody (MG1131) of the IgG4 isotype was employed in this study. This antibody was designed to specifically block the TIGIT immune checkpoint pathway by minimizing Fc-mediated effector functions, thereby enabling precise evaluation of the effects of TIGIT blockade. Experimental data demonstrated that both TIGIT blockade and PD-1 blockade enhanced the tumor-killing activity of CTLs to a comparable extent. This indicates that TIGIT plays a critical role in the direct regulation of effector T cell function, and its blockade may restore immune responses by reversing T cell exhaustion. The primary mechanisms involved are likely as follows: Relief of Co-stimulatory Suppression: TIGIT competitively binds to its ligand PVR (CD155) with high affinity, thereby inhibiting the activation of the co-stimulatory receptor CD226. MG1131 blocks the TIGIT-PVR interaction, consequently liberating the CD226-mediated PI3K/Akt activation signal, which directly enhances CTL function. Synergy with the PD-1 Pathway: TIGIT is frequently co-expressed with PD-1 on exhausted T cells. Dual blockade may produce a synergistic effect, not only promoting the expansion of tumor-antigen-specific T cells but also preventing their differentiation into a terminally exhausted state, thereby maintaining a more potent effector cell pool. Modulation of the Immune Microenvironment: TIGIT is highly expressed on regulatory T cells (Tregs) and is associated with their suppressive function. Targeting TIGIT may attenuate Treg-mediated suppression or induce functional “fragility,” contributing to the remodeling of the immunosuppressive tumor microenvironment. However, TIGIT’s expression patterns, functional regulation, and interactions with other immune checkpoint molecules in different tumor types remain incompletely understood. Future research must deepen mechanistic insights into TIGIT signaling in HCC and optimize combinations with other immunotherapies (e.g., peptide vaccines and CAR-T cell therapy) to achieve more precise and effective tumor immunotherapy.

Despite significant anti-tumor efficacy in xenograft mouse models, clinical translation faces challenges. For example, long-term toxicity and immune-related adverse events of vorinostat and TIGIT blockade require further evaluation in clinical trials [31, 32]. Elucidation of MAEL antigen presentation mechanisms, Vorinostat’s regulation of other genes, and TIGIT blockade’s pleiotropic effects remain ongoing priorities.

## Conclusions

This study establishes a novel HCC immunotherapy strategy by combining MAEL peptide vaccination, HDACi vorinostat induction, and TIGIT blockade, representing a major advancement in hepatocellular carcinoma treatment and providing a new paradigm for HCC immunotherapy. Future steps will include launching multicenter clinical trials of MAEL-DC vaccine combined with vorinostat and tiragolumab, with particular focus on immune reconstitution and viral reactivation risks in HBV-associated patients. Integration of artificial intelligence-driven antigen screening and synthetic biology technologies (e.g., CAR-T cells targeting MAEL) aims to achieve precision and personalized breakthroughs in HCC therapy.

## Data Availability

Not applicable.
